# Trends and Factors Associated With the Mortality Rate of Depressive Episodes: An Analysis of the CDC Wide-Ranging Online Data for Epidemiological Research (WONDER) Database

**DOI:** 10.7759/cureus.41627

**Published:** 2023-07-10

**Authors:** Radhey Patel, Abimbola E Arisoyin, Obiaku U Okoronkwo, Shaw Aruoture, Okelue E Okobi, Mirian Nwankwo, Emeka Okobi, Francis Okobi, Oshoriamhe Elisha Momodu

**Affiliations:** 1 Psychiatry and Behavioral Sciences, Avalon University School of Medicine, Willemstad, CUW; 2 Internal Medicine, College of Medicine, University of Lagos, Lagos, NGA; 3 School of Medicine, Kwame Nkrumah University of Science and Technology, Kumasi, GHA; 4 Psychiatry, Behavioral Hospital of Bellaire, Houston, USA; 5 Family Medicine, Medficient Health Systems, Laurel, USA; 6 Family Medicine, Lakeside Medical Center, Belle Glade, USA; 7 Neonatology, Peter Lougheed Centre, Alberta Health Services, Alberta, CAN; 8 Dentistry, Ahmadu Bello University Teaching Hospital, Abuja, NGA; 9 Research and Development, Covance, Hanover, USA; 10 Family Medicine, Crimea State Medical University, Simferopol, RUS

**Keywords:** cdc wonder, major depressive disorder, trend analysis, mortality rate, depressive episode

## Abstract

Background

Depressive episodes are associated with increased mortality rates across the United States. Recognizing the relationship between depression and physical health, understanding the contributing factors, and addressing disparities are critical in reducing mortality rates and improving the overall well-being of individuals experiencing depressive episodes. Continued research, public health efforts, and collaborative approaches are essential to tackle this complex public health concern effectively. Studying the mortality rate trends of depressive episodes along with other related factors will help enhance the understanding of the condition, which, in turn, will assist in reducing mortality rates in the vulnerable population.

Methodology

Data from the CDC Wide-Ranging Online Data for Epidemiologic Research (WONDER) database on the Underlying Cause of Death were examined to identify individuals who experienced fatal outcomes related to depressive episodes from 1999 to 2020. The WONDER database refers to the online system used by the CDC to make its various resources accessible to the public and public health experts. CDC WONDER offers access to a broader range of information on public health.

Results

A total of 13,290 individuals who died from depressive episodes between 1999 and 2020 were identified. Data analysis revealed an overall mortality rate of 0.20 per 100,000 individuals during the specified period. The highest mortality rates were observed in the years 2003 (0.28), 2001 (0.27), and 1999 (0.27). The analysis revealed significant disparities in mortality rates among different demographic groups. Older adults, females, specific racial groups, including Whites and African Americans, and specific geographic areas, including the Midwest, Northeast, South, and West, exhibited higher mortality rates associated with depressive episodes.

Conclusions

The study identified that older individuals, females, Whites, and African Americans, as well as certain geographic regions, exhibited an increased likelihood of mortality related to depressive episodes. These findings highlight the importance of understanding the complex interplay between mental health and mortality. The findings emphasize the importance of addressing disparities in mental health outcomes among different demographic groups. Identifying vulnerable populations can inform targeted interventions and resources to address the elevated mortality risk.

## Introduction

Depressive episodes are associated with increased mortality rates across the United States. Recognizing the relationship between depression and physical health, understanding contributing factors, and addressing disparities are critical in reducing mortality rates and improving the overall well-being of individuals experiencing depressive episodes. Continued research, public health efforts, and collaborative approaches are essential to tackle this complex public health concern [1,2].

A depressive episode is a prolonged duration of extreme despondency, accompanied by a dearth of interest in or delight in various activities and an array of physical and emotional symptoms with significant effects on an individual’s everyday life. The condition is considered indicative of major depressive disorder (MDD), which is a widespread and severe mental health disorder. Individuals experiencing a depressive episode may persistently feel sadness, hopelessness, or emptiness. They may lose interest in previously enjoyed activities, have difficulty concentrating or making decisions, and experience changes in appetite and sleep patterns [1-3].

In 2008, MDD was ranked by the World Health Organization (WHO) as the third major cause of the global disease burden. The WHO predicts that by 2030, MDD will become the primary cause. A significant portion of the US population is affected by MDD [4]. Furthermore, the National Institute of Mental Health (NIMH) estimates that nearly 20.6 million adults in the United States, almost 8.4% of the US adult population, have experienced at least one severe depressive episode as of 2019 [5]. A Substance Abuse and Mental Health Services Administration study revealed that nearly 7.2% of the US adult population experienced a major depressive episode (MDE) in 2018. As a result, the National Health Interview Survey found that approximately 3.5% of deaths at the population level were linked to depression or anxiety [6-9].

In the United States, depressive episodes or MDD are significant concerns for public health. While depressive episodes can manifest at any age, their prevalence rates vary across different age groups. Although depression can affect individuals of all ages, it is most commonly observed during adolescence or early adulthood. Additionally, data drawn from the National Health Interview Survey has revealed that in the United States, younger adults aged between 18 and 25 years reported the highest prevalence rate (17%) of severe depressive episodes in 2019, followed closely by adults aged between 26 and 49 years at 9.1%, and individuals aged 50 years and above at 5.4%. Research suggests that females are more prone to experiencing depressive episodes compared to males. This gender disparity may be influenced by biological, hormonal, psychosocial, and cultural factors [5,9].

Depressive episodes can harm individuals’ everyday functioning, social connections, and overall well-being. They can disrupt work, education, and personal relationships and, in severe cases, increase the risk of suicide [10]. Unfortunately, many people who experience depression fail to seek or receive appropriate treatment. This reluctance may be influenced by stigma, limited access to mental health services, lack of awareness, and failure to recognize symptoms as indicators for seeking help [11,12-16].

Several studies have examined the association between depression and mortality, but the relationship remains complex and multifaceted. This analysis aims to explore the existing secondary data in the CDC Wide-Ranging Online Data for Epidemiologic Research (WONDER) database on the association between depression and mortality, considering various factors such as age, gender, comorbidities, and socioeconomic status. We can comprehensively understand this relationship and its implications for public health by synthesizing these findings.

## Materials and methods

Study sample and data source

The researchers queried the Underlying Cause of Death database from the CDC WONDER database for individuals who succumbed to depressive episodes from 1999 to 2020. The CDC WONDER database is a web-based system developed by the CDC in the United States. It provides access to a wide range of public health data and information. The CDC WONDER database is a secondary source of publicly available mortality data obtained from death certificates. It offers information about the decedents’ place of death alongside specific demographics, such as the cause of death, demographic factors, and other relevant details. The present study did not require informed consent and ethical committee approval, given that the data utilized had no identifiable information and was publicly accessible through the National Center for Health Statistics data use contract. Data analysis was performed for the study between May 10 and May 21, 2023.

The CDC WONDER database is valuable for accessing various health-related data, including mortality rates. The mortality rate describes the overall number of deaths in a given population over a certain period. Typically, mortality rates are expressed as the number of deaths per 100,000 or 1,000 persons in a given population. Consequently, cause-specific mortality rates and all-cause mortality rates concerning the main conditions and disorders were investigated through the utilization of the International Classification of Diseases 10th Revision (ICD-10) codes F32.0, which relates to minor depressive episodes; F32.1, which relates to mild depressive episodes; and F32.2, which relates to acute depressive episodes without psychotic symptoms. Furthermore, the researchers utilized ICD-10 codes F32.3, which entails acute depressive episodes that present with psychotic symptoms; F32.8, which involves various other depressive episodes; and F32.9, which entails depressive episodes with unspecified symptoms.

Data analysis

Mortality rate data were stratified by gender and across the years, and race was categorized according to the US Census Bureau: African Americans/Blacks, non-Hispanic (NH), Asians and Hispanics, White, and Pacific Islanders (API). The categorization of mortality rates by location included various divisions within the United States, such as the South Atlantic, West North Central, Mountain, New England, Pacific, East North Central, West South Central, Mid-Atlantic, and East South Central divisions. It also included mortality rates for each of the 50 states, the four census regions (South, Northeast, West, and Midwest), and the 10 regions defined by the Health and Human Services (HHS). The aggregate data for the selected period (1999-2020), including the patient attributes concerning the mortality rate per 100,000 people and the total number of deaths over the years for all patients, were summarized. Notably, only available patients’ data were used in every model, and appropriate sample sizes were used in every figure and table. No modifications were made for the available data and multiple correlations. The latest version of Excel (2019) was used to conduct all statistical analyses.

## Results

We utilized CDC WONDER’s Underlying Cause of Death database to acquire aggregate data on 13,290 individuals who died from a depressive episode from 1999 to 2020. The overall mortality rate per 100,000 people was found to be 0.20 from 1999 to 2020, with the highest mortality rates recorded in 2003 (0.28), 2001 (0.27), and 1999 (0.27) (see Table 1 below).

**Table 1:** Year-wise mortality rate trend for demographic characteristics (1999-2020). M = Million; total population = approximate total US population This table was an original concept of the authors.

		Years and corresponding variables																					
	Variables	1999	2000	2001	2002	2003	2004	2005	2006	2007	2008	2009	2010	2011	2012	2013	2014	2015	2016	2017	2018	2019	2020
Overall data	Total number of deaths	760	713	773	753	821	704	663	623	563	557	528	569	536	534	520	505	484	511	538	510	495	630
	Total population	279M	281M	285M	288M	290M	293M	296M	298M	301M	304M	307M	309M	312M	314M	316M	319M	321M	323M	326M	327M	328M	329M
	Overall mortality rate	0.27	0.25	0.27	0.26	0.28	0.24	0.22	0.21	0.19	0.18	0.17	0.18	0.17	0.17	0.16	0.16	0.15	0.16	0.17	0.16	0.15	0.19
	95% CI	0.25-0.29	0.23-0.27	0.25-0.29	0.24-0.28	0.26-0.3	0.22-0.26	0.21-0.24	0.19-0.23	0.17-0.2	0.17-0.2	0.16-0.19	0.17-0.2	0.16-0.19	0.16-0.18	0.15-0.18	0.14-0.17	0.14-0.16	0.14-0.17	0.15-0.18	0.14-0.17	0.14-0.16	0.18-0.21
	Standard error	0.01	0.01	0.01	0.01	0.01	0.01	0.01	0.01	0.01	0.01	0.01	0.01	0.01	0.01	0.01	0.01	0.01	0.01	0.01	0.01	0.01	0.01
Gender	Female	0.34	0.34	0.36	0.37	0.39	0.32	0.3	0.29	0.23	0.23	0.22	0.23	0.22	0.22	0.21	0.2	0.2	0.2	0.2	0.19	0.19	0.24
	Male	0.2	0.17	0.18	0.15	0.17	0.16	0.15	0.13	0.14	0.14	0.13	0.13	0.12	0.12	0.11	0.11	0.1	0.12	0.13	0.12	0.12	0.14
Race	White	0.32	0.29	0.31	0.31	0.33	0.28	0.27	0.24	0.22	0.21	0.20	0.22	0.21	0.2	0.2	0.19	0.18	0.19	0.2	0.19	0.18	0.22
	Black or African American	0.06	0.08	0.09	0.08	0.09	0.06	0.04	0.08	0.04	0.07	0.04	0.05	0.04	0.06	0.03	0.05	0.06	0.07	0.06	0.06	0.05	0.08
	Asian or Pacific Islander	-	-	-	-	-	-	-	-	-	-	0.06	-	-	0.07	-	-	-	-	-	0.05	-	0.06
Age	35-44 years	0.04	-	0.05	-	0.02	-	-	-	-	0.03	-	0.03	-	-	-	-	-	-	-	-	-	-
	45-54 years	0.04	0.06	0.04	0.05	0.06	0.06	0.05	0.04	0.05	0.04	0.06	0.05	0.03	0.03	0.03	0.05	0.05*	0.06	0.06	0.05	0.03	0.06
	55-64 years	0.10	0.08	0.08	0.09	0.10	0.07	0.05	0.08	0.10	0.12	0.08	0.10	0.10	0.11	0.09	0.08	0.11	0.12	0.14	0.09	0.14	0.16
	65–74 years	0.35	0.30	0.36	0.32	0.27	0.36	0.21	0.22	0.22	0.24	0.22	0.28	0.24	0.28	0.27	0.23	0.28	0.28	0.29	0.30	0.27	0.36
	75–84 years	2.07	1.65	1.89	1.93	1.85	1.56	1.73	1.57	1.22	1.15	1.14	1.16	1.03	1.09	0.89	0.94	0.82	0.88	0.95	0.77	0.82	0.98
	85+ years	8.86	9.06	9.34	8.90	10.43	8.43	7.33	6.64	5.83	5.39	4.94	5.06	5.00	4.31	4.49	4.01	3.59	3.51	3.32	3.51	2.97	3.75
Hispanic origin	Hispanic or Latino	0.04	0.03	0.04	0.04	0.04	0.04	0.03	0.02	0.03	0.03	-	0.03	0.03	0.03	0.03	0.03	0.03	-	0.03	0.04	0.04	0.05
	Not Hispanic or Latino	0.03	0.04	0.04	0.04	0.03	0.03	0.03	0.03	0.03	0.03	0.20	0.21	0.20	0.20	0.19	0.18	0.18	0.19	0.20	0.18	0.18	0.22

Mortality rate trends analysis

Based on Location

The analysis revealed significant geographic variations in mortality rates associated with depressive episodes. Table 2 below presents mortality rate trend data based on location for four census regions, nine census divisions, 10 HHS regions, and mortality rates in the top five US states and counties for 1999-2020.

**Table 2:** Mortality rate trend based on location (1999-2020). The correlation coefficient between the variables “Number of deaths” and “Mortality Rate per 100,000” is approximately -0.6209. The acronyms represent states in the United States of America: Alabama: AL; Alaska: AK; Arizona: AZ; Arkansas: AR; California: CA; Colorado: CO; Connecticut: CT; Delaware: DE; Florida: FL; Georgia: GA; Hawaii: HI; Idaho: ID; Illinois: IL; Indiana: IN; Iowa: IA; Kansas: KS; Kentucky: KY; Louisiana: LA; Maine: ME; Maryland: MD; Massachusetts: MA; Michigan: MI; Minnesota: MN; Mississippi: MS; Missouri: MO; Montana: MT; Nebraska: NE; Nevada: NV; New Hampshire: NH; New Jersey: NJ; New Mexico: NM; New York: NY; North Carolina: NC; North Dakota: ND; Ohio: OH; Oklahoma: OK; Oregon: OR; Pennsylvania: PA; Rhode Island: RI; South Carolina: SC; South Dakota: SD; Tennessee: TN; Texas: TX; Utah: UT; Vermont: VT; Virginia: VA; Washington: WA; West Virginia: WV; Wisconsin: WI; Wyoming: WY

Variables		Number of deaths	Mortality rate per 100,000
Census region	Census region 1: Northeast	2,707	0.22
	Census region 2: Midwest	4,190	0.29
	Census region 3: South	3,831	0.15
	Census region 4: West	2,562	0.16
Census division	Division 1: New England	1,040	0.33
	Division 2: Middle Atlantic	1,667	0.19
	Division 3: East North Central	2,562	0.25
	Division 4: West North Central	1,628	0.36
	Division 5: South Atlantic	2,098	0.16
	Division 6: East South Central	672	0.17
	Division 7: West South Central	1,061	0.13
	Division 8: Mountain	868	0.18
	Division 9: Pacific	1,694	0.16
HHS region	HHS region #1 CT, ME, MA, NH, RI, VT	1,040	0.33
	HHS region #2 NJ, NY	955	0.15
	HHS region #3 DE, DC, MD, PA, VA, WV	1,312	0.20
	HHS region #4 AL, FL, GA, KY, MS, NC, SC, TN	2,170	0.16
	HHS region #5 IL, IN, MI, MN, OH, WI	3,018	0.27
	HHS region #6 AR, LA, NM, OK, TX	1,142	0.14
	HHS region #7 IA, KS, MO, NE	1,020	0.34
	HHS region #8 CO, MT, ND, SD, UT, WY	601	0.25
	HHS region #9 AZ, CA, HI, NV	1,108	0.11
	HHS region #10 AK, ID, OR, WA	924	0.33
County*	Marshall County, MS	31	3.91
	Grand Traverse County, MI	31	1.63
	Josephine County, OR	25	1.38
	Douglas County, OR	23	0.98
	Humboldt County, CA	24	0.82
State*	Vermont	85	0.62
	North Dakota	87	0.57
	Oregon	435	0.52
	New Hampshire	134	0.47
	Iowa	296	0.44

The correlation coefficient of approximately -0.6209 suggests a moderate negative correlation between the number of deaths and the mortality rate per 100,000 individuals. This indicates that, on average, higher numbers of deaths tend to be associated with lower mortality rates, and lower numbers of deaths tend to be associated with higher mortality rates.

**Figure 1:**
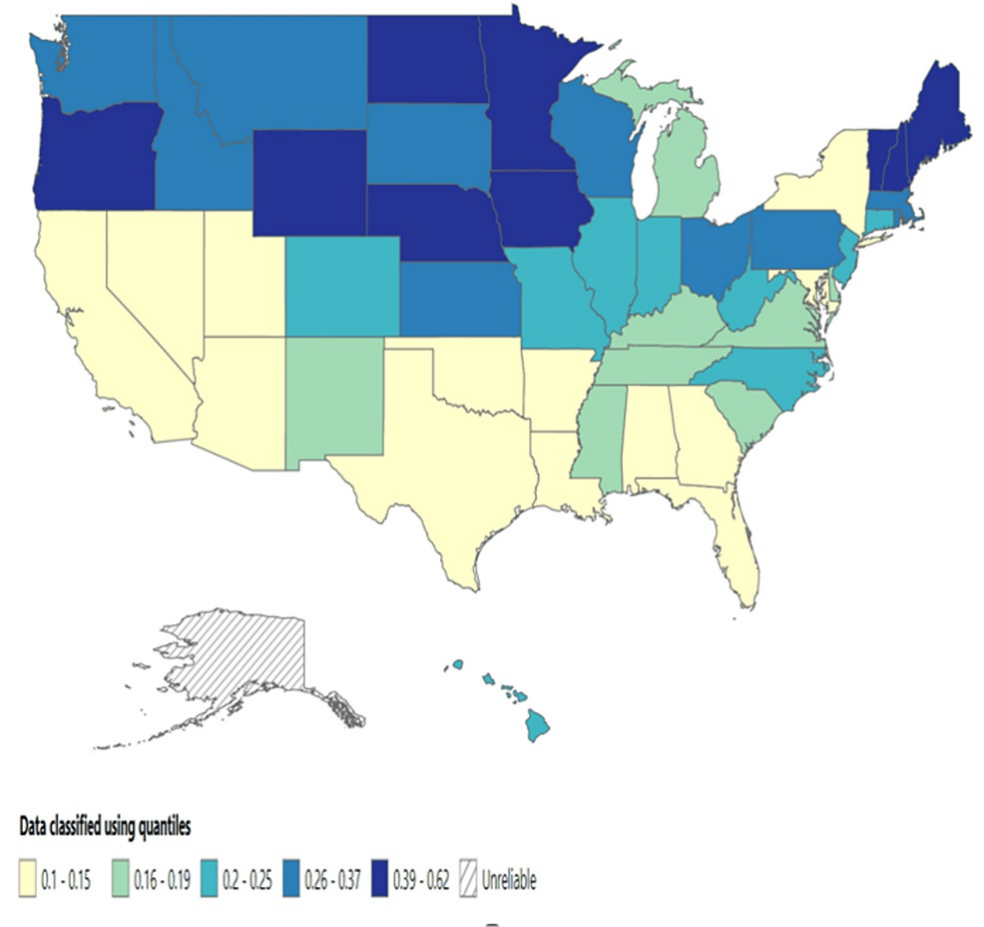
Mortality rate across the US states (1999-2020).

**Figure 2:**
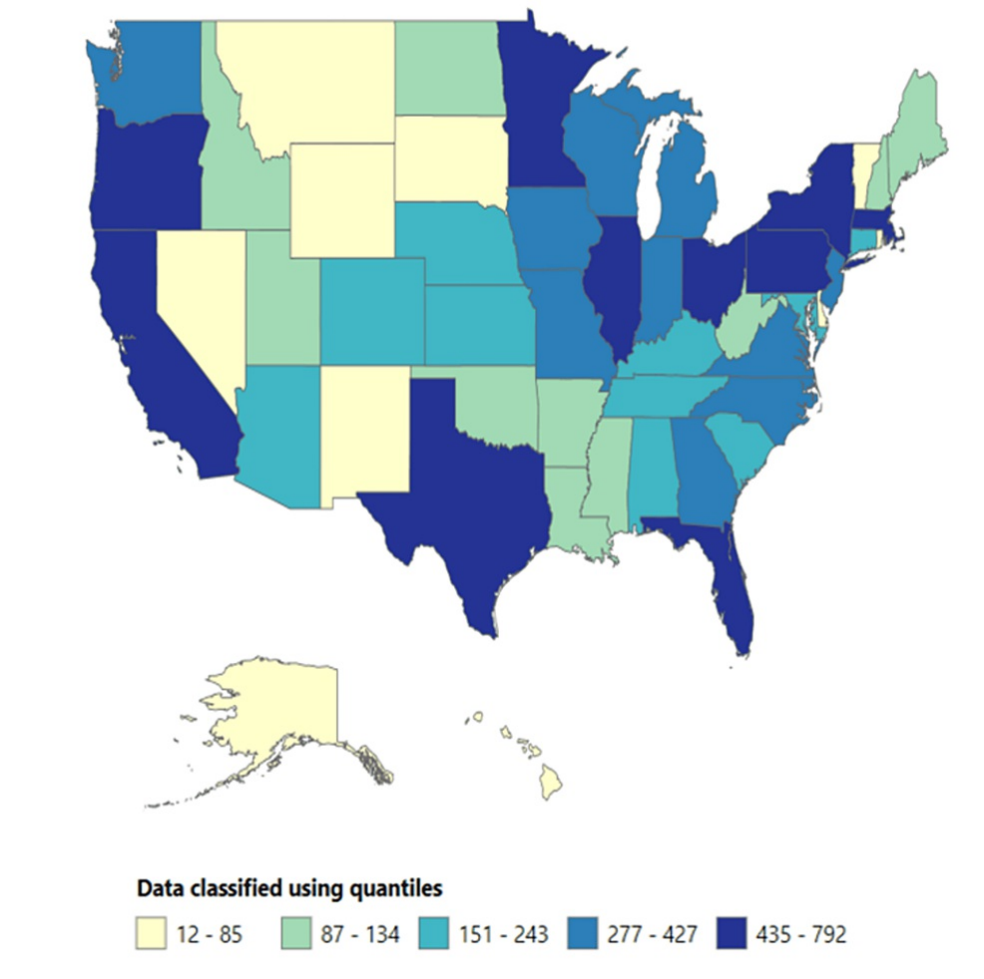
Total number of deaths across the US states (1999-2020).

**Figure 3:**
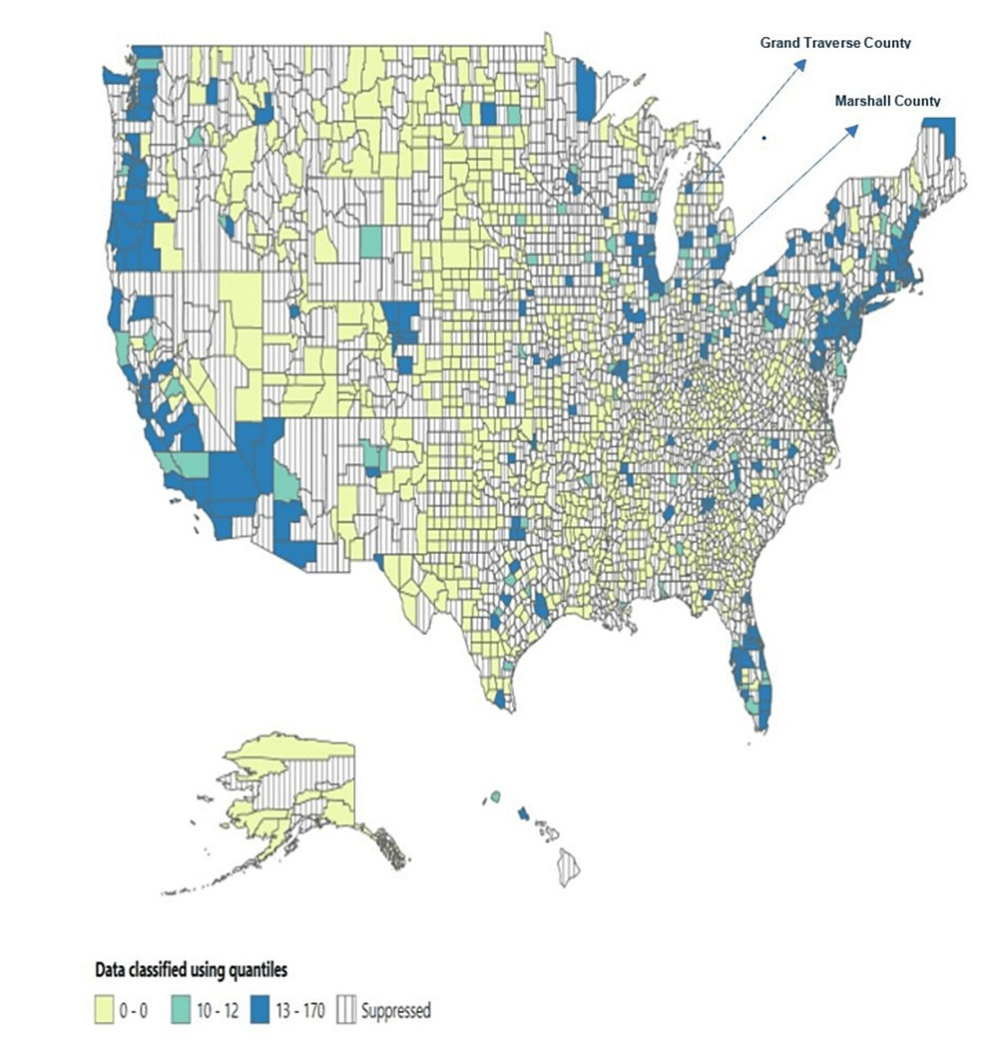
Total number of deaths across the US counties (1999-2020).

**Figure 4:**
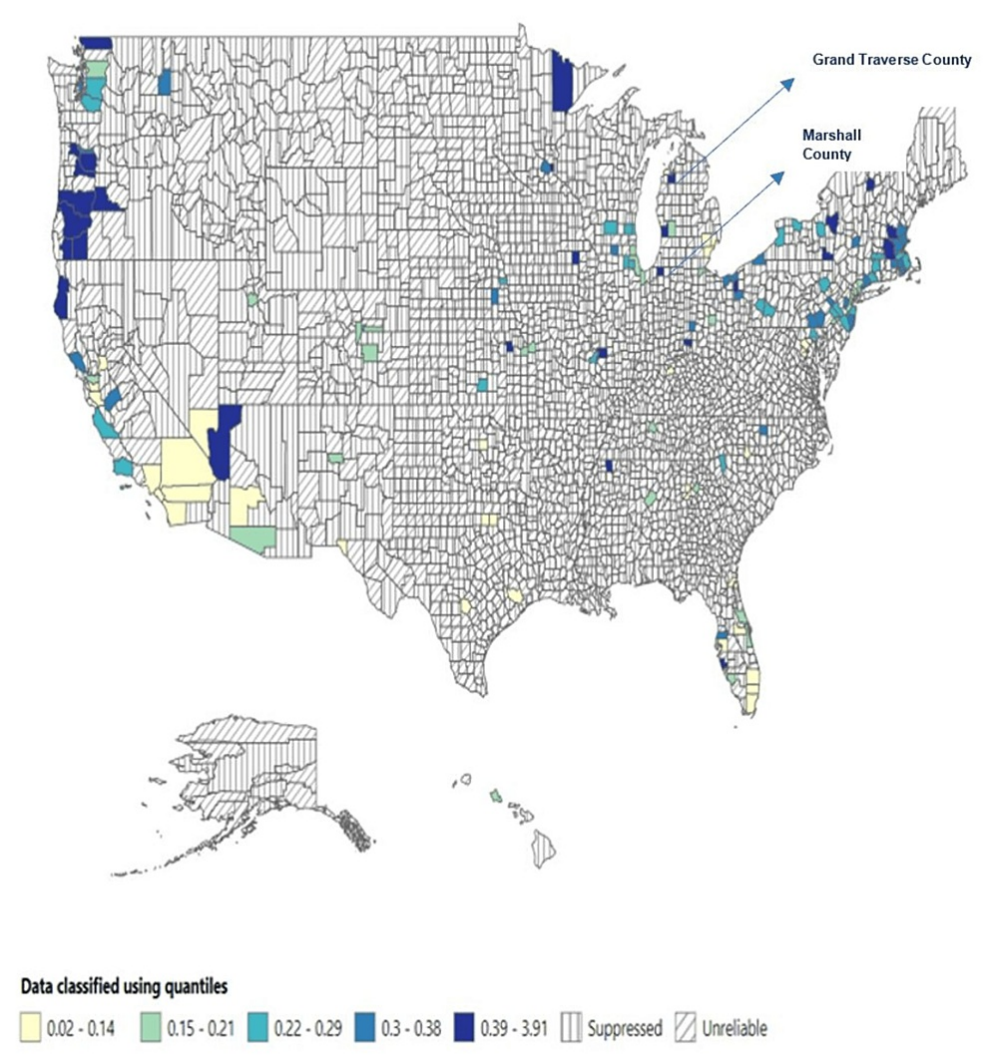
Mortality rate across the US counties (1999-2020).

The highest mortality rate was reported in Vermont (0.62 per 100,000 people), followed by North Dakota (0.57 per 100,000 people) and Oregon (0.52 per 100,000 people). In comparison, Marshall County and Grand Traverse County reported the highest mortality rates, 3.91 and 1.63 per 100,000 people, respectively (see Figures 1-4 below).

Based on Gender

**Figure 5:**
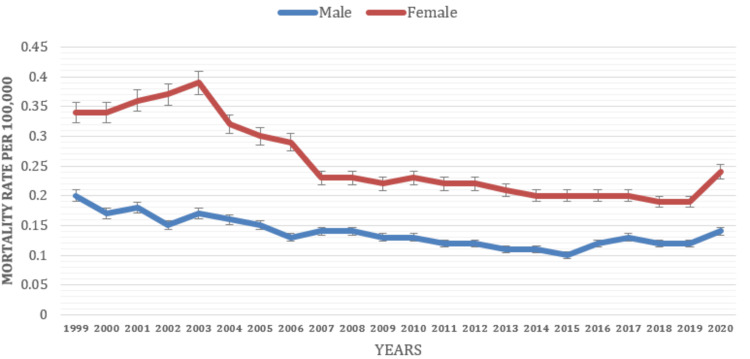
Mortality rate based on gender classification (1999-2021).

The study found significant gender disparities in mortality rates attributed to depressive episodes, with males and females exhibiting distinct patterns and levels of mortality associated with these episodes. Females generally showed higher mortality rates (0.25) associated with depressive episodes than males (0.12). This indicates that males may have a relatively lower mortality risk than females when experiencing depressive episodes (see Figure 5 below).

Based on Race

**Figure 6:**
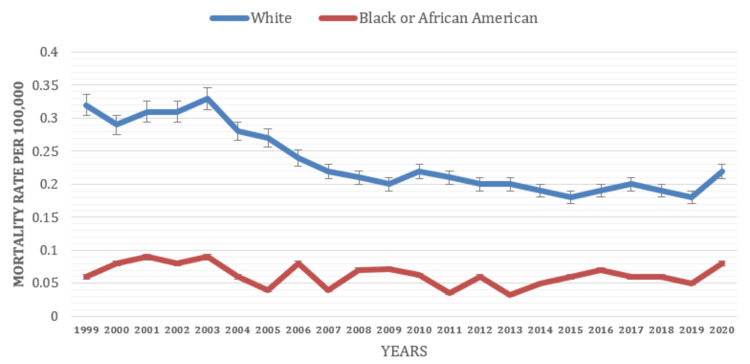
Mortality rate based on race (1999-2021).

The analysis identified racial groups with higher mortality rates associated with depressive episodes. The study found that individuals from White racial groups exhibited higher mortality rates than other racial groups. Based on racial classification, the White racial group had an average mortality rate of 0.23 compared to the rate of 0.06 for the Black or African American racial group (Figure 6).

Based on Age

**Figure 7:**
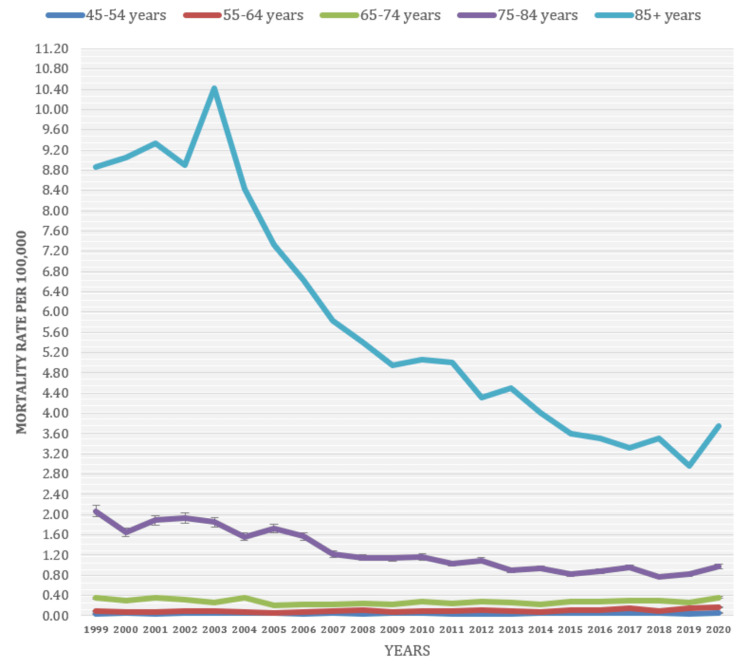
Mortality rate age classification (1999-2021).

The study found substantial variations in mortality rates linked to depressive episodes among different age groups, with individuals aged 85 and above consistently exhibiting higher mortality rates than younger age groups. The average mortality rate of 5.85 was recorded in patients above 85 years of age, followed by 1.28 in those between 74 and 84 years of age. The study also identified transition age groups, such as individuals in their middle ages (45-54), where mortality rates associated with depressive episodes showed varying patterns. These age groups exhibited intermediate mortality rates, falling between the higher rates observed in older adults and the lower rates seen in younger age groups (see Figure 7 below).

## Discussion

Extensive research has consistently demonstrated that individuals with mental illness experience higher mortality rates than those without such conditions, although these studies were not exclusively focused on depression [16-27,28]. This study identified significant variations in mortality rates across different regions. For instance, Vermont reported the highest mortality rate (0.62 per 100,000 people), followed by North Dakota (0.57 per 100,000 people) and Oregon (0.52 per 100,000 people). Further research is needed to address the regional differences in mortality rates associated with depressive episodes.

The correlation coefficient, ranging from -1 to 1, indicates the strength and direction of the relationship between two variables. In this case, the correlation coefficient of approximately -0.6209 suggests a moderate negative correlation between the number of deaths and the mortality rate per 100,000. This indicates that as the number of deaths increases, the mortality rate per 100,000 tends to decrease and vice versa. It is important to note that the strength of the relationship is not extremely strong, suggesting that other factors may also influence the relationship between these variables. Additionally, the negative sign of the correlation coefficient indicates the inverse direction of the relationship. This means that as the number of deaths increases, the mortality rate per 100,000 tends to decrease, and as the number of deaths decreases, the mortality rate per 100,000 tends to increase. It is crucial to remember that correlation does not imply causation. The correlation coefficient solely measures the statistical relationship between the variables, and other factors may be responsible for the observed pattern.

The study reported disparities in mortality rates related to depressive episodes based on gender, with higher rates observed in females and lower rates in males. These results underscore the necessity for gender-specific approaches in mental health care, suicide prevention, and support systems to address the distinct challenges both males and females face. However, it is crucial to recognize that even lower mortality rates do not diminish the seriousness and impact of depressive episodes on the overall health and well-being of males. This further highlights the significance of providing sufficient mental health support and resources for both men and women to alleviate the burden of mortality associated with depressive episodes. In contrast to these findings, other single-center studies, including meta-analysis, have found that depression-related excess mortality is higher in men than women [16,27-29]. However, some other contrasting previous studies demonstrated a higher prevalence of MDEs among adult females compared to males [5,9,17,18].

The study uncovered notable disparities in mortality rates associated with depressive episodes among different racial groups. The analysis revealed variations in mortality rates based on race, suggesting that race plays a role in influencing the risk of mortality linked to depressive episodes. This variation in mortality rates among depressed patients has remained controversial over the years, with different studies reporting varying statistics [19,21,23,25,30,31]. The analysis also considered the potential impact of social determinants of health on observed racial disparities in mortality rates. Socioeconomic status, healthcare accessibility, discrimination, and cultural factors can all contribute to the varying risk of mortality associated with depressive episodes among different racial groups. In our study, the average mortality rate for the White race was 0.23, whereas, for the Black or African American population, it was 0.06. These findings were also consistent with a previously published study that found a more substantial effect of baseline depressive symptoms on the long-term risk of all-cause mortality in Whites versus Blacks [19-21]. However, regarding symptoms and prevalence, these findings contrasted with a previous study that reported higher depressive symptomatology among racial/ethnic minorities compared to Whites. On the contrary, a retrospective cohort study by Parikh et al. noted a significant disparity in mortality rates between African Americans and Caucasians. The study revealed a significant interaction between race and depression (p < 0.001). The analysis demonstrated that the impact of newly developed depression on overall mortality was more pronounced among African Americans (adjusted hazard ratio (aHR) = 1.32; 95% confidence interval (CI) = 1.26-1.38; p < 0.001) compared to Caucasians (aHR = 1.15; 95% CI = 1.07-1.24; p < 0.001) [30,31]. Further research is needed to understand the complex interactions between race, social determinants, and mortality rates [22-24].

The study observed higher mortality rates linked to depressive episodes in older people. Patients above 85 years old had an average mortality rate of 5.85, while those between 74-84 years old had a rate of 1.28. This suggests that older adults face a higher mortality risk than younger individuals, who exhibit comparatively lower rates. These findings highlight the potential vulnerability of older adults to the negative consequences associated with depressive episodes. However, in contrast to these results, a cohort study conducted by Oude et al. found that depressive disorder is associated with increased mortality in people under 60 [9,25-27].

On the other hand, a comprehensive study conducted by Craig et al., examining the mortality rates of individuals with mental health conditions across eight states in the United States, showed that public mental health clients consistently exhibited a significantly higher relative risk of mortality compared to the general populations of their respective states. Disturbingly, the deceased public mental health clients passed away at considerably younger ages, resulting in the loss of several decades of potential life compared to their living counterparts nationwide [28].

It is important to acknowledge the limitations of the study. The analysis relied on available data from the CDC WONDER database, which might not capture all relevant factors influencing mortality rates in depressive episodes. Additionally, the study did not explore potential underlying causes or factors contributing to the observed racial disparities in mortality rates. Future research should delve deeper into these factors to better understand the relationship between race, mortality rates, and depressive episodes. Another limitation is that the accuracy of diagnosing depressive episodes may vary across healthcare providers and settings. The CDC WONDER database relies on the accuracy of diagnostic coding and reporting, which can be influenced by factors such as clinician training, diagnostic criteria changes over time, and individual variation in interpretation. Misdiagnosis or underdiagnosis of depressive episodes can impact the reliability of the analysis.

One strength of our study is its ability to compare and benchmark across different locations, demographic groups, and periods. This comparative analysis aids in identifying disparities, assessing the efficacy of interventions, and guiding evidence-based decision-making in treating depressive episodes and associated mortality rates. The database contains population-level data, providing insights into mortality rates associated with depressive episodes at a population level rather than relying solely on individual case studies or small-scale research. This representative nature of the data enhances the applicability and relevance of the findings to public health planning and policy development. However, it is worth noting that the analysis presented specific limitations, including diagnostic precision and data availability. These limitations should be considered when interpreting and drawing conclusions based on the study’s findings.

## Conclusions

The study identified certain factors that were associated with increased mortality rates and revealed significant discrepancies in mortality rates based on factors such as age, gender, race, and location. The examined mortality rates from 1999 to 2020 and revealed several noteworthy findings. Overall, the mortality rate per 100,000 people during this period was 0.20. The highest mortality rates were recorded in 2003 (0.28), 2001 (0.27), and 1999 (0.27). Analyzing mortality rates by geographic regions, it was found that Vermont had the highest rate at 0.62 per 100,000 people, followed by North Dakota (0.57 per 100,000 people) and Oregon (0.52 per 100,000 people). In terms of gender, the study observed that females exhibited higher mortality rates (0.25) associated with depressive episodes compared to males (0.12). Examining mortality rates by race, the study discovered that individuals from White racial groups displayed higher mortality rates than other racial groups. Lastly, the study explored mortality rates by age. It consistently found that individuals aged 85 and above exhibited higher mortality rates than younger age groups. Overall, the study shed light on the different factors influencing mortality rates, including periods, geographic regions, gender, race, and age. These findings contribute to our understanding of mortality patterns and can help inform strategies for targeted interventions and healthcare planning.
